# WDFY4 Promotes the Progression of Atherosclerosis by Regulating Ferroptosis Mediated by the LAPTM5/CDC42/mTOR/4EBP1/SLC7A11 Pathway

**DOI:** 10.1111/jcmm.70729

**Published:** 2025-08-03

**Authors:** Nier Zhong, Xiting Nong, Guang Yang

**Affiliations:** ^1^ Department of Cardiology Shaanxi Provincial People's Hospital Xi'an Shaanxi China; ^2^ Department of Endocrinology Xi'an Central Hospital Xi'an Shaanxi China

**Keywords:** atherosclerosis, CDC42/mTOR/4EBP1/SLC7A11 pathway, ferroptosis, LAPTM5, WDFY4

## Abstract

Atherosclerosis (AS) is a prevalent chronic inflammatory cardiovascular disease and leading cause of death worldwide. WD repeat and FYVE domain‐containing 4 (WDFY4), a susceptibility gene for autoimmune diseases and reported AS hub gene, is associated with ferroptosis and inflammation. However, the mechanism by which WDFY4 affects atherosclerosis remains unclear. Here, WDFY4 was upregulated in oxidised low‐density lipoprotein (ox‐LDL)‐treated cells and high‐fat diet (HFD)‐fed ApoE^−/−^ mice. Endothelium‐specific transgenic mice were employed to disrupt the expression level of WDFY4 in vivo. WDFY4 knockdown inhibited ox‐LDL‐induced ferroptosis, cell injury and inflammation in HAECs in vitro. The STRING database predicted lysosomal transmembrane protein 5 (LAPTM5) as a WDFY4‐interacting partner, validated by co‐immunoprecipitation (Co‐IP) and immunofluorescence co‐localisation. Mechanistically, WDFY4 interference inhibited LAPTM5 expression and activated the downstream CDC42/mTOR/4EBP1/SLC7A11 pathway. LAPTM5 overexpression or ML141 (a CDC42 inhibitor) rescued the WDFY4 knockdown‐mediated inhibition of ferroptosis, cell damage and inflammation in model cells. WDFY4 or LAPTM5 knockdown reduced plaque formation, lipid deposition and pro‐inflammatory factors in vivo. Finally, mice with endothelial‐specific WDFY4 knockout were protected against atherosclerosis. Thus, WDFY4 affects ferroptosis‐related AS via the LAPTM5/CDC42/mTOR/4EBP1/SLC7A11 pathway. These findings identify a novel role for WDFY4 in AS and its potential as a therapeutic target.

## 1 Introduction

Atherosclerosis (AS), a chronic inflammatory cardiovascular disease, develops via dysregulated lipid metabolism, maladaptive immune responses and disrupted redox homeostasis [1, 2]. Its pathogenesis is driven by metabolic risk factors including dyslipidemia, type 2 diabetes, obesity and nonalcoholic fatty liver disease [2]. AS formation involves inflammatory reactions, endothelial damage and lipid deposition [3], with characteristic histopathological features encompassing endothelial injury, inflammatory cell infiltration, vascular smooth muscle cell proliferation and lipid accumulation. Notably, functional alterations and dysregulated cell death in vascular cells critically influence plaque formation and stability, thereby affecting progression [4]. Therefore, identifying novel therapeutic targets and elucidating the precise molecular mechanisms underlying AS initiation and development is critical.

Ferroptosis, an iron‐dependent regulated cell death mechanism, is characterised by reactive oxygen species (ROS) accumulation and lethal lipid peroxides (LPO) [5, 6]. This process manifests distinct morphological features, including cellular swelling and mitochondrial ultrastructural abnormalities—specifically cristae reduction/destruction, increased membrane density, outer membrane rupture and plasma membrane integrity loss [7, 8, 9]. Ferroptosis involves intracellular iron overload, glutathione (GSH) depletion, amino acid metabolic dysregulation, lipid oxidation and impaired glutathione peroxidase 4 (GPX4) function [10, 11, 12]. Emerging evidence links atherosclerotic pathogenesis to iron dyshomeostasis, excessive ROS generation and reduced GPX4 activity [13]. Despite these associations, the precise regulatory network connecting ferroptosis to AS progression remains incompletely clear, necessitating further mechanistic investigation.

WD Repeat‐and FYVE Domain‐Containing Protein 4 (WDFY4), a member of the BEACH (Beige and Chediak‐Higashi) domain‐containing protein family and the fourth member of the WDFY family, conserved across multiple species [14, 15], comprises 3184 amino acids and contains putative functional domains including WD40 and BEACH. WDFY4 protein is encoded by WDFY4 on chromosome 10q11.23 [16]. WDFY4 is implicated as a susceptibility locus in autoimmune disorders such as systemic lupus erythematosus [17], rheumatoid arthritis [18] and clinically amyopathic dermatomyositis [19]. It plays crucial roles in dendritic cell cross‐presentation and reactive oxygen species‐induced CD8+ T cell apoptosis [20, 21, 22]. Additionally, WDFY4 modulates B cell differentiation and is linked to atypical autophagy processes and ferroptosis in B lymphocytes [17, 23]. Bioinformatic analyses indicate WDFY4 upregulation in atherosclerosis samples compared to healthy controls, designating it as a hub gene in AS pathogenesis [24]. Nevertheless, its functional contributions to atherosclerotic progression and underlying molecular mechanisms remain poorly characterised.

In this study, we focus on the atherosclerosis‐associated hub gene WDFY4 to investigate its functional role in AS pathogenesis. Our findings demonstrate that WDFY4 modulates ferroptosis‐driven AS progression by regulating the LAPTM5/CDC42/mTOR/4EBP1/SLC7A11 signalling pathway.

## 2 Materials and Methods

### 2.1 Cell Culture and Ox‐LDL Treatment

Human aortic endothelial cells (HAECs, Punosai Biotechnology) were maintained in ECM medium supplemented with 10% foetal bovine serum (FBS), 1% penicillin/streptomycin and 1% endothelial cell growth supplement, under standard culture conditions (37°C, 5% CO_2_). Medium replacement was performed at 50%–60% confluence. To establish in vitro AS models, cells were treated with increasing ox‐LDL concentrations (0, 25, 50, 100, 150, 200 μg/mL) for 6, 12, 24 and 48 h to determine the optimal ox‐LDL treatment concentration and time.

### 2.2 Cell Transfection

Lentiviral vectors encoding shRNA constructs (sh‐WDFY4 and sh‐LAPTM5) were designed, synthesised and packaged by Shanghai Sangon. The LAPTM5 overexpression vector was constructed by Guangzhou Yunzhou. Transient transfections were performed using Lipofectamine 2000 reagent (Thermo Fisher Scientific) following the manufacturer's protocol, with subsequent experiments conducted 48 h post‐transfection.

### 2.3 RT‐qPCR

Total RNA was extracted from HAECs and mouse aortic tissue using TRIzol reagent (Thermo Fisher). cDNA synthesis was performed with a PrimeScript RT reagent kit (TaKaRa, Dalian) according to the manufacturer's instructions. SYBR Green Premix Pro Taq HS qPCR Kit was then used for qPCR. The qPCR conditions were 95°C for 10 min, 35 cycles, 95°C for 15 s, 60°C for 20 s, 72°C for 15 s. GAPDH was used as an internal reference; the relative expression calculation formula was 2^−ΔΔCt^, and the primer sequence was as follows: WDFY4 (forward: 5′‐AAA GGC TGG CAG AAG ATG TG‐3′, reverse: 5′‐ATC CTG ATC CGC GTC ACT C‐3′), TNF‐α (forward: 5′‐GGT GCC TAT GTC TCA GCC TCT T‐3′, reverse: 5′‐GCC ATA GAA CTG ATG AGA GGG AG‐3′), IL‐1β (forward, 5′‐GCA ACT GTT CCT GAA CTC AAC T‐3′, reverse: 5′‐ATC TTT TGG GGT CCG TCA ACT‐3′) and GAPDH (forward 5′‐TGG ATT TGG ACG CAT TGG TC‐3′, reverse 5′‐TTT GCA CTG GTA CGT GTT GAT‐3′). The designed primers were sent to Bioengineering (Shanghai) Co. Ltd. for synthesis.

### 2.4 Western Blotting

According to the manufacturer's instructions, total protein was extracted from HAECs and mouse aortic tissue using the mammalian protein extraction kit (Thermo Fisher). Proteins were separated by SDS‐PAGE, transferred to PVDF membrane (Millipore, Billerica, MA, USA) and blocked with 5% skimmed milk powder (1 g skimmed milk powder: 20 mL 1 × TBST reagent) for 2 h. Subsequently, membranes were incubated overnight at 4°C with primary antibodies: WDFY4 (1: 500, orb512258, Biorbyt, UK), LAPTM5 (1: 1000, PA5‐23585, Thermo Fisher), GPX4 (1: 500, ab125066, Abcam, UK), SLC7A11 (1: 500, ab307601, Abcam), ACSL4 (1: 500, ab155282, Abcam), CDC42 (1: 500, ab187643, Abcam), mTOR (1:500, ab134903, Abcam), 4EBP1 (1: 500, ab32024, Abcam) and GAPDH (1: 1000, ab181602, Abcam). After washing, membranes were incubated with secondary antibody (1: 500, ab205718, Abcam) at room temperature for 2 h. Finally, Pierce ECL Western Blotting Substrate was used to visualise the protein [25].

### 2.5 CCK‐8

CCK‐8 kit (C0039, Beyotime) was used to detect cell viability. HAECs were seeded in 96‐well plates at a density of 2 × 10^3^ cells per well. After the cells were cultured for 0, 24, 48 and 72 h, 10 μL CCK‐8 reagent was added. Following 1 h incubation, absorbance was measured at 450 nm.

### 2.6 Immunofluorescence

HAECs in the logarithmic growth phase were centrifuged at 1000 r/min for 5 min, washed with PBS, and smeared onto slides. Cells were fixed (4% paraformaldehyde), permeabilised (0.1% Tween‐20, 10 min), washed (PBS × 3) and blocked (1% BSA/PBS, pH 7.5, 30 min). The samples were incubated overnight at 4°C with the primary antibodies anti‐WDFY4 or anti‐LAPTM5. Subsequently, they were incubated with the secondary antibody IgG at room temperature for 1 h in the dark. After that, neutral resin was added dropwise for sealing. Finally, the samples were observed under a fluorescence microscope, and ImageJ software was used for analysis. For immunofluorescence staining of mouse vascular tissue, tissues were fixed with 4% PFA for 10 min and permeabilised with 0.3% Triton X‐100 with PBS for another 10 min. The subsequent steps and cell staining were consistent.

### 2.7 Detection of Fe^2+^ Content

HAECs in the logarithmic growth phase were taken and centrifuged at 3000 rpm for 10 min to remove particles and polymers. The cell supernatant was collected, and Fe^2+^ levels in the cells were analysed using a Fe^2+^ ELISA kit (abs580234, abs580234, China).

FerroOrange fluorescence probe (Dojindo, Kumamoto, Japan) was used to assess the cellular levels of free iron (Fe^2+^). Briefly, after indicated treatment, HAECs were incubated with 200 μL cell culture in serum‐free culture media containing 1 μM FerroOrange at 37°C in a humidified atmosphere containing 5% CO_2_ for 30 min. The cells were observed, and images were captured using a confocal microscope (Olympus, Japan). The fluorescence intensity was quantified using a multimode plate reader (Ex: 543 nm, Em: 580 nm).

### 2.8 ROS Level Detection

HAECs were seeded in 6‐well plates at a concentration of 2 × 10^5^ cells/well and cultured overnight at 37°C in a 5% CO_2_ incubator. Subsequently, cells were incubated with 10 μM DCFH‐DA fluorescent probe (HY‐D0940, MedChemExpress, USA) at 37°C, 5% CO_2_ for 30 min, and then washed with PBS. Additionally, to detect the level of ROS in vivo, fresh aortic sinus sections were incubated with 10 μM DCFH‐DA in PBS at 37°C for 30 min. After incubation, the sections were washed three times consecutively with PBS to remove the residual probe. The fluorescence intensity was observed by a Zeiss fluorescence microscope, and the relative fluorescence intensity was calculated by Image J software to represent the intracellular ROS level.

### 2.9 Cell Death Detection

HAECs were collected and centrifuged at 1000 r/min for 5 min. PBS buffer was added to the cells, and the cell number was adjusted to 10^6^/mL. 100 μL of the cell suspension was taken, and 10 μL of PI reagent (Vazyme Biotech, Jiangsu, China) was added to the cell suspension and mixed well. The cell suspension was placed at 4°C in the dark for 5 min and then added to a glass slide. Subsequently, the cells were observed and photographed using a fluorescence microscope, and the percentage of PI‐positive cells was used to represent dead cells.

### 2.10 Detection of Oxidative Stress Indicators and Endothelial Vasodilator Factors

The concentration of malondialdehyde (MDA) and the level of GSH in HAECs were detected using an MDA detection kit (S0131M, Biyuntian) and a GSH detection kit (ml076450, Shanghai enzyme—linked biology), respectively.

For the determination of nitric oxide (NO) production and endothelial nitric oxide synthase (eNOS) activity in the media of HAECs, a nitrate reductase NO kit (no. A012) and endothelial nitric oxide synthase Assay Kit (no. H195) from Nanjing Jiancheng Bioengineering Institute (Nanjing, China) were used according to the manufacturer's protocol. Endothelin‐1 (ET‐1) was measured using an ET‐1 detection kit (no. E—EL‐ H0064c; Elabscience Biotechnology Co. Ltd., Wuhan, China).

### 2.11 Transmission Electron Microscopy (TEM) Assay

Fresh tissues or cells were fixed in 2.5% electron microscope‐grade glutaraldehyde fixing solution (G1102; Servicebio, Wuhan, China) for 1 h at room temperature. Subsequently, they were incubated overnight at 4°C. After fixation with 0.1% osmium tetroxide, the samples underwent dehydration and embedding processes to prepare ultrathin sections. These sections were then stained with lead citrate and uranyl acetate. Finally, TEM images were captured using a transmission electron microscope (JEM—1400 PLUS, Tokyo, Japan).

### 2.12 Co‐Immunoprecipitation Analysis (Co‐IP)

HAECs in the logarithmic phase were collected and lysed using radioimmunoassay precipitation buffer. The lysed cells were centrifuged at 14000 r/min at 4°C for 15 min, and the supernatant was collected. Subsequently, anti‐DFY4 and IgG or anti‐APTM5 and IgG were added and incubated with protein A/G magnetic beads on a shaker at 4°C for 2 h. Finally, the expression of WDFY4 and LAPTM5 proteins in the two systems was detected by Western blotting.

### 2.13 Experimental Mice and Treatment

8‐week‐old male C57BL/6 mice (body weight 18–22 g) and 8‐week‐old male ApoE^−/−^ mice (body weight 18–22 g) with C57BL/6 background were purchased from Beijing Vital River Laboratory Animal Technology Co. Ltd. The C57BL/6 mice served as the blank control group and were fed a normal diet. ApoE^−/−^ mice were fed a high‐fat diet (Research Diets, no. D12108C; 20% fat and 1.25% cholesterol) and injected with sh‐WDFY4, sh‐LAPTM5, sh‐NC or empty lentiviral vectors every other day.

For WDFY4 knockout experiments, C57BL/6 WDFY4^fl/fl^ mice were crossed with C57BL/6 Cdh5‐Cre^ERT2^ mice to generate an inducible endothelial‐specific Wdfy4 knockout model (Wdfy4^ECKO^). WDFY4^fl/fl^ mice (referred to as WDFY4^WT^ mice) in the same litter were used for controls. All mice (WDFY4^CKO^ and WDFY4^fl/fl^ littermate controls) received 2 mg tamoxifen/d for 5 consecutive days via intraperitoneal injection beginning at 5 weeks of age. To induce atherosclerosis, all mice received a single dose of AAV8‐Pcsk9D377Y (100 μ; volume containing 3 × 10^11^ virus particles/mouse) at 7 weeks of age [26]. All mice were fed HFD from 8 weeks of age for 16 weeks. All mice were maintained under specific‐pathogen‐free conditions. After anaesthesia with 2.5% isoflurane gas, blood was collected from the mice. The mice were euthanised to remove the organs and tissues around the aorta, fully exposing the aortic arch to the bifurcation of the abdominal aorta, and intercepting the proximal end of the aorta. This study was approved by the Ethics Committee of Shaanxi Provincial People's Hospital (Approval number: 2023 K‐S147). Animal experiments follow the Guidelines for the Care and Use of Laboratory Animals.

### 2.14 Detection of Serum Lipid Profile and Inflammatory Factors

After anaesthesia with 2.5% isoflurane gas, blood was collected from the mice. Serum was obtained by centrifuging the blood at 4°C, 1000 g, for 10 min and stored at −80°C for lipid panel analysis. Lipid levels, including serum total cholesterol (TC; no. A111‐1‐1), triglycerides (TG; no. A110‐1‐1), high‐density lipoprotein cholesterol (HDL‐C; no. A112‐1‐1) and low‐density lipoprotein cholesterol (LDL‐C; no. A113‐1‐1), were measured using detection kits from Nanjing Jiancheng Bioengineering Institute (Nanjing, China).

The concentrations of TNF‐α, IL‐6 and IL‐1β in mice serum and in the cultural supernatant were measured by using ELISA Kits (Abcam, Cambridge, MA) following the company's directions.

### 2.15 Oil Red O Staining

Aortic sections were fixed in 10% formalin and incubated in 1,2‐propanediol for 2 min. Then, they were immersed in oil red O dye solution for 20 min at room temperature. The sections were re‐tained with haematoxylin for 2–3 min and rinsed with running water to turn the nucleus blue. The sections were then sealed with 50% PBS glycerol, and images were observed and collected under a light microscope. Ten mice were randomly selected from each treatment group [27].

### 2.16 H&E Staining

After formaldehyde fixation, haematoxylin staining, differentiation with 1% hydrochloric acid ethanol, 0.5% eosin staining and dehydration treatment, the aortic sections were sealed with neutral gum. Images were then observed and collected under a light microscope.

### 2.17 Data Analysis

SPSS 22.0 software and GraphPad Prism 8.0.2 were employed to analyse the data. Student's t‐test, one‐way analysis of variance (ANOVA), or two‐way ANOVA were used to compare the quantitative indexes of different treatment groups. The data were expressed as Means ± SD. Significance levels were defined as *p < 0.05 and **p < 0.01.

## 3 Results

### 3.1 WDFY4 Is Upregulated in AS Model Cells

**FIGURE 1:**
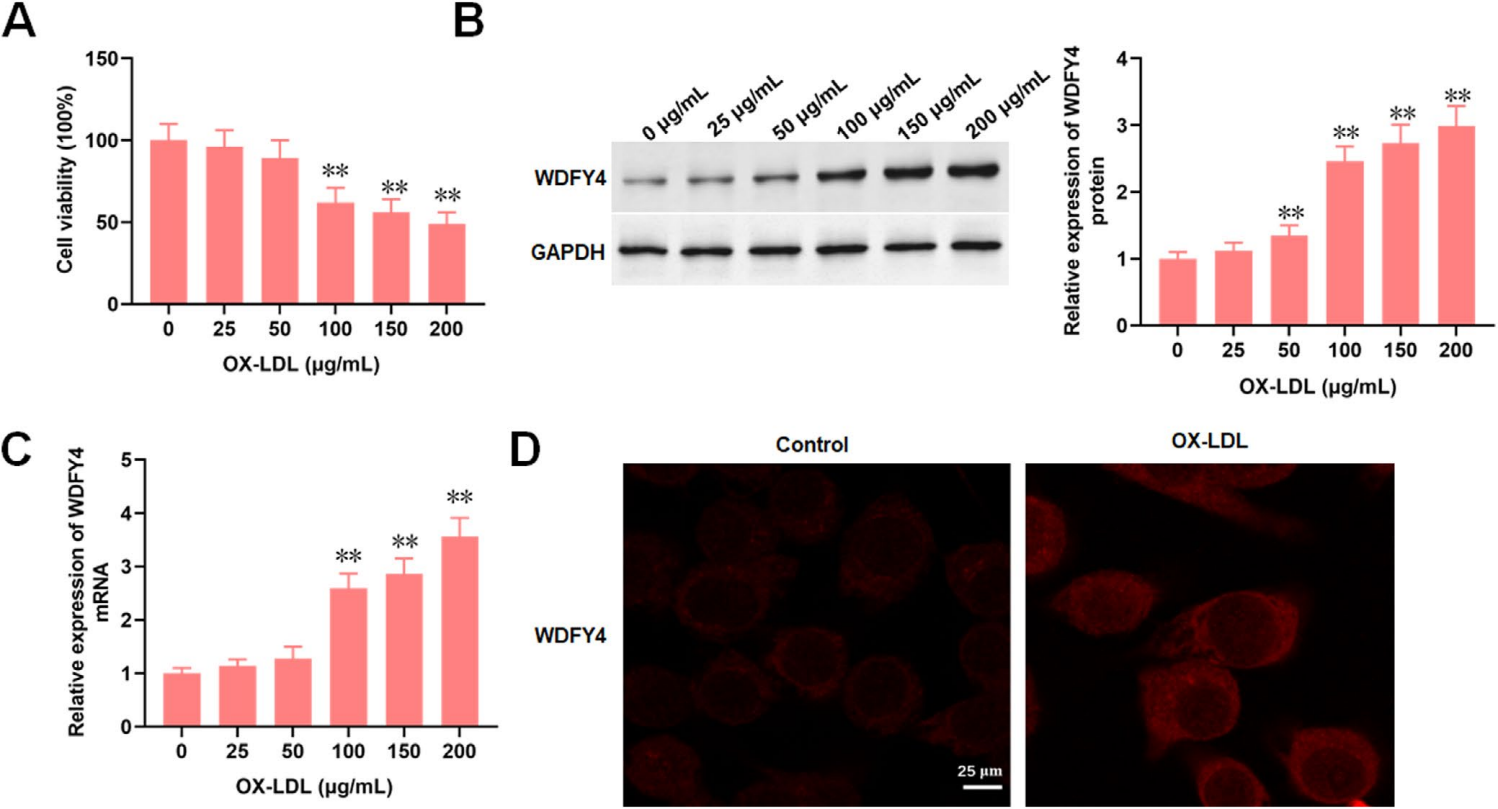
WDFY4 is up‐regulated in ox‐LDL‐treated HAEC and inhibits cell viability. HAECs were treated with 0 μg/mL, 25 μg/mL, 50 μg/mL, 100 μg/mL, 150 μg/mL and 200 μg/mL ox‐LDL for 24 h. (A) The effects of different concentrations of ox‐LDL on the activity of HAEC were detected. (B) The expression level of WDFY4 protein in HAEC treated with different concentrations of ox‐LDL was detected. (C) The expression of WDFY4 mRNA in HAEC treated with different concentrations of ox‐LDL was detected. (D) The expression of WDFY4 was detected by immunofluorescence. Scale bar, 25 μm. *n* = 4. One‐way ANOVA was used for comparison between multiple groups. Data are presented as mean ± SD. *p < 0.05 and **p < 0.01.

Ox‐LDL was employed to simulate oxidative stress‐induced endothelial injury in atherosclerosis. In this study, a series of time‐ and dose‐effect experiments were conducted to validate the responsiveness of HAECs to ox‐LDL and establish an optimal dosage for subsequent assays. As shown in the Figure S1, HAECs treated with ox‐LDL (0, 25, 50, 100, 150 and 200 μg/mL) for 12 and 24 h demonstrated a dose‐dependent decrease in cell viability, accompanied by significant upregulation of WDFY4 protein expression at a range from 50 to 200 μg/mL, with the levels of WDFY4 plateauing above 100 μg/mL (Figure S1A,B). HAECs treated with ox‐LDL (0, 50, 100 or 200 μg/mL) over an extended timeframe (6, 12, 24 and 48 h) showed a significant increase in WDFY4 expression at 24 h, plateauing above 24 h (Figure S1C,D). Based on these findings, 100 μg/mL ox‐LDL exposure for 24 h was selected for establishing the oxidative injury model in HAECs. Additionally, immunofluorescence staining showed that ox‐LDL treatment significantly enhanced WDFY4 fluorescence intensity in HAECs (Figure 1D).

### 3.2 Knockdown of WDFY4 Inhibits Ox‐LDL‐Induced Ferroptosis and Inflammation in HAECs

**FIGURE 2:**
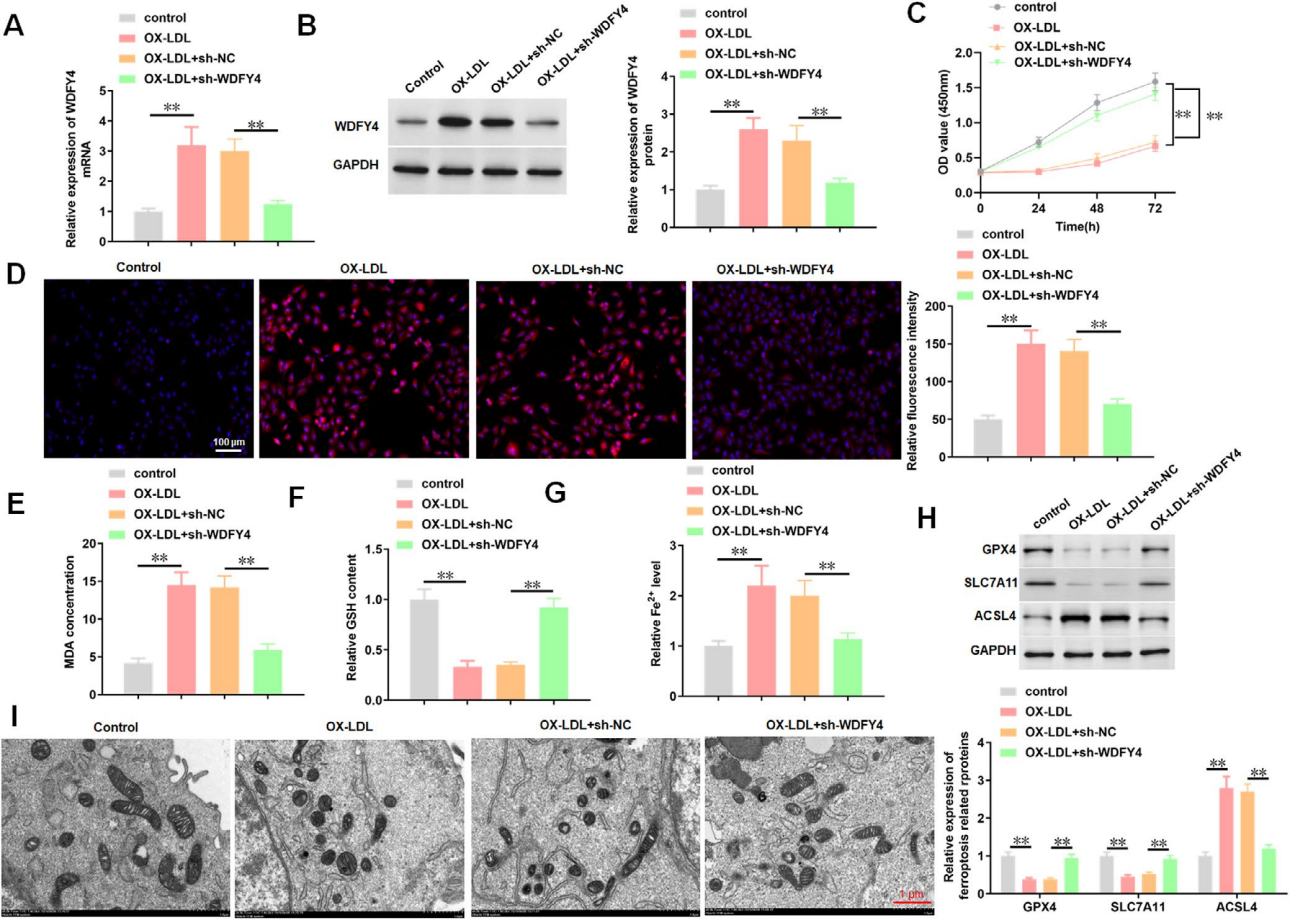
Knockdown of WDFY4 inhibits ox‐LDL‐induced ferroptosis in HAECs. After HAECs were treated with 100 μg/mL ox‐LDL for 24 h, HAECs were transfected with sh‐WDFY4. (A) RT‐qPCR was used to detect the expression level of WDFY4 mRNA in HAECs. (B) Western blotting was used to detect the expression level of WDFY4 protein in HAECs. (C) CCK‐8 was used to detect the activity of HAECs. (D) The level of lipid ROS in HAECs was detected by DCFH‐DA fluorescent probe method. Scale bar, 100 μm. (E) MDA content was detected with MDA assay kit. (F) GSH level was detected with GSH assay kit. (G) ELISA was used to detect Fe^2+^ content in HAECs. (H) Western blotting was used to detect the level of ferroptosis‐related proteins. (I) Representative transmission electron microscopy pictures of mitochondria ultrastructure in HAECs were showed. Scale bar, 1 μm. *n* = 4. One‐way ANOVA or Two‐way ANOVA was used for comparison between multiple groups. **p < 0.01.

Previous studies have reported that WDFY4 serves as a hub gene in AS [24] and is associated with ferroptosis [23]. Thus, we focus on the role of WDFY4 in ferroptosis‐associated AS. qPCR and Western blotting results showed that WDFY4 was highly expressed in ox‐LDL‐treated HAECs, and WDFY4 knockdown inhibited the expression of WDFY4 in ox‐LDL‐treated HAECs (Figure 2A,B). CCK‐8 assays indicated that ox‐LDL significantly reduced HAEC viability, whereas WDFY4 interference alleviated this effect (Figure 2C). Knockdown of WDFY4 reversed ox‐LDL‐induced increases in lipid peroxidation markers, including lipid ROS content (Figure 2D) and MDA production (Figure 2E), while elevating GSH levels in HAECs (Figure 2F). Further studies revealed that the increase of Fe^2+^ levels induced by ox‐LDL treatment in HAECs, as determined by ELISA (Figure 2G) and Ferro Orange probe (Figure S2), were also reversed by WDFY4 knockdown. In addition, ox‐LDL treatment increased the expression level of ACSL4 in HAECs, and decreased the expression levels of GPX4 and SLC7A11, while these effects caused by ox‐LDL could be reversed by WDFY4 knockdown (Figure 2H). We also observed the ultrastructure of mitochondria by TEM. TEM revealed that ox‐LDL induced mitochondrial shrinkage and cristae disruption in HAECs, whereas WDFY4 knockdown significantly ameliorated mitochondrial damage (Figure 2I). These findings suggest that WDFY4 induces ferroptosis in HAECs.

**FIGURE 3:**
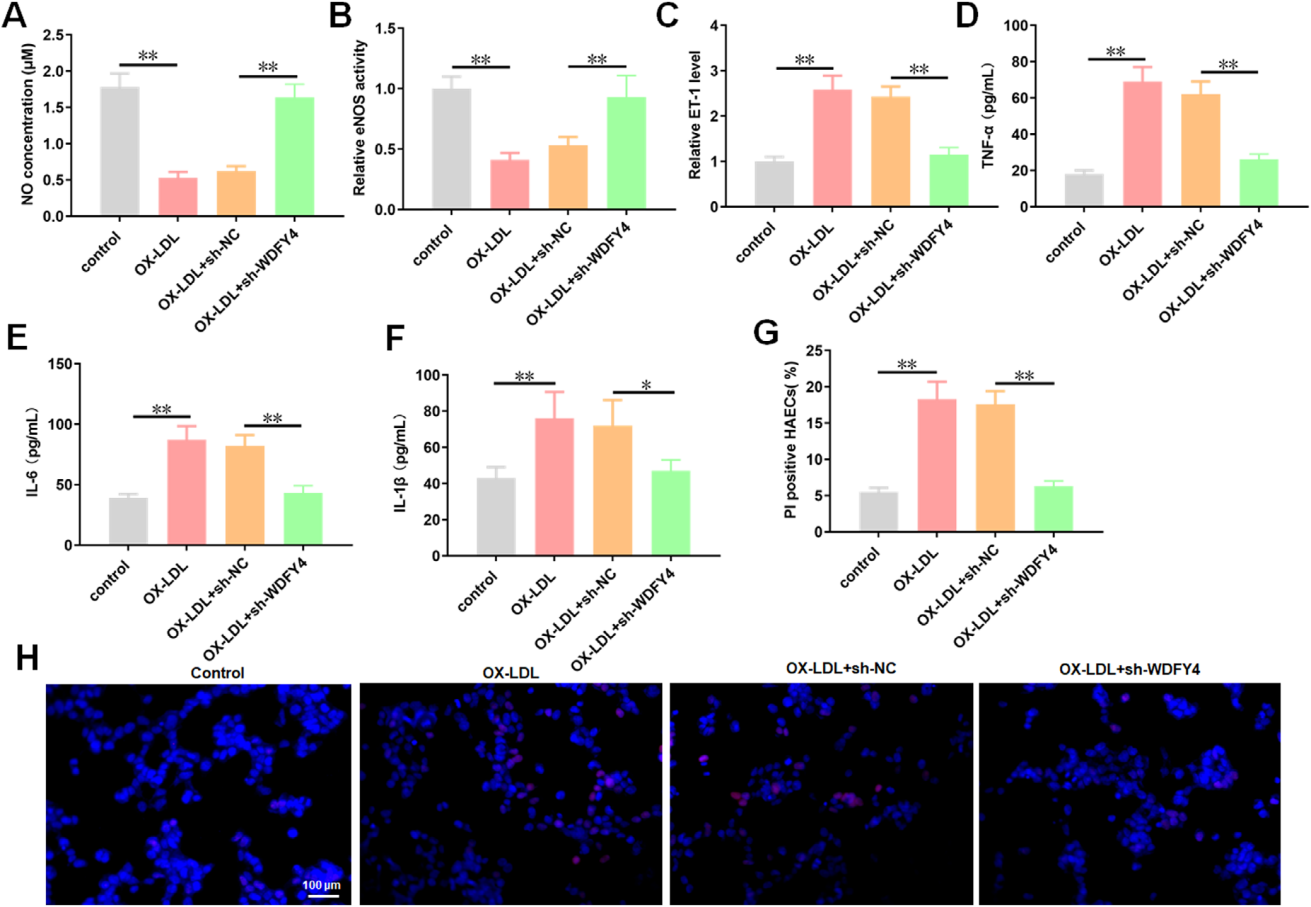
Interference with WDFY4 reduces ox‐LDL induced HAEC cell death and inflammatory response. After HAECs were treated with 100 μg/mL ox‐LDL for 24 h, HAECs were transfected with sh‐WDFY4. (A) The concentration of endothelial cell function marker NO in the supernatant of HAEC cells was detected by NO assay kit. (B) The activity of endothelial cell function marker eNOS in HAECs supernatant was detected by ELISA. (C) The level of endothelial cell function marker ET‐1 in the supernatant of HAECs was detected by ELISA. (D–F) The secretion level of inflammatory factors (TNF‐α, IL‐6 and IL‐1β) in the supernatant of HAECs was detected by ELISA. (G, H) PI staining was used to detect the mortality of HAECs treated with ox‐LDL. Scale bar, 100 μm. *n* = 4. One‐way ANOVA was used for comparison between multiple groups. *p < 0.05, **p < 0.01.

Endothelial injury is a primary pathological factor underlying the development of AS. Thus, we investigated the impact of WDFY4 on endothelial cell injury. We examined the effects of WDFY4 knockdown on endothelial cell function markers and the levels of inflammatory factors. The results indicated that treatment with ox‐LDL decreased the NO concentration (Figure 3A), inhibited eNOS activity (Figure 3B) and elevated the ET‐1 level (Figure 3C). Conversely, interference with WDFY4 mitigated the effects of ox‐LDL on these endothelial cell function markers (Figure 3A–C). For inflammatory factors, ox‐LDL enhanced the secretion of TNF‐α, IL‐6 and IL‐1β in HAEC supernatants, whereas WDFY4 knockdown suppressed their release (Figure 3D–F). Furthermore, PI staining was employed to analyse the effect of WDFY4 on HAEC death. The results demonstrated that ox‐LDL increased the percentage of PI‐positive cells, which was attenuated by administration of sh‐WDFY4 (Figure 3G,H). Collectively, these results indicate that WDFY4 knockdown alleviates ox‐LDL‐induced HAEC injury and inflammatory responses.

### 3.3 WDFY4 and LAPTM5 Co‐Expressed in HAECs

**FIGURE 4:**
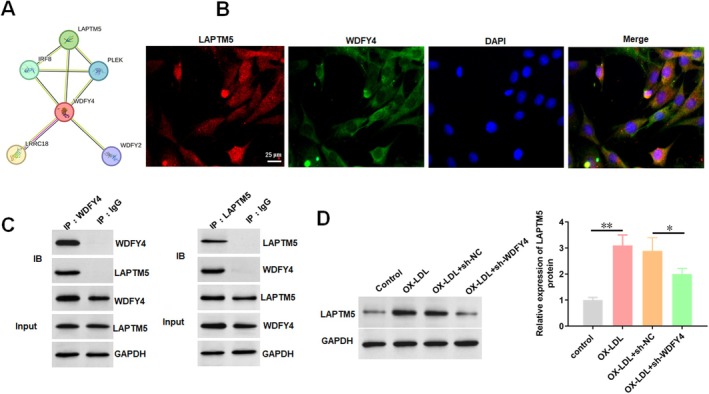
WDFY4 interacts with LAPTM5 to promote LAPTM5 expression. After HAECs were treated with 100 μg/mL ox‐LDL for 24 h, HAECs were transfected with sh‐WDFY4. (A) WDFY4 interacting proteins were predicted. (B) Immunofluorescence staining of WDFY4 and LAPTM5 was showed. Scale bar, 25 μm. (C) Co‐IP assay was used to verify the interaction between WDFY4 and LAPTM5. (D) The effect of WDFY4 knockdown on the expression of LAPTM5 protein in HAEC was detected by Western blotting. *n* = 4. One‐way ANOVA was used for comparison between multiple groups. *p < 0.05, **p < 0.01.

To elucidate the molecular mechanism by which WDFY4 regulates ferroptosis in HAECs, the STRING database was used to predict potential interacting partners of WDFY4 (Figure 4A). Among the candidate proteins, LAPTM5 was prioritised due to its known association with autophagy‐related atherosclerosis [28]. The interaction between WDFY4 and LAPTM5 was verified by Co‐IP assay and immunofluorescence co‐localisation. Immunofluorescence microscopy revealed their co‐localisation in HAECs (Figure 4B), and Co‐IP assays demonstrated physical association between WDFY4 and LAPTM5 (Figure 4C). Notably, Western blotting showed that ox‐LDL treatment significantly upregulated LAPTM5 expression in HAECs, and this induction was markedly attenuated by WDFY4 knockdown (Figure 4D). These findings establish the WDFY4–LAPTM5 interaction as a novel regulatory axis in ox‐LDL‐mediated cellular responses.

### 3.4 WDFY4 Interacts With LAPTM5 and Promotes Ferroptosis in HAECs by Inhibiting CDC42/mTOR/4EBP1/SLC7A11 Pathway

**FIGURE 5:**
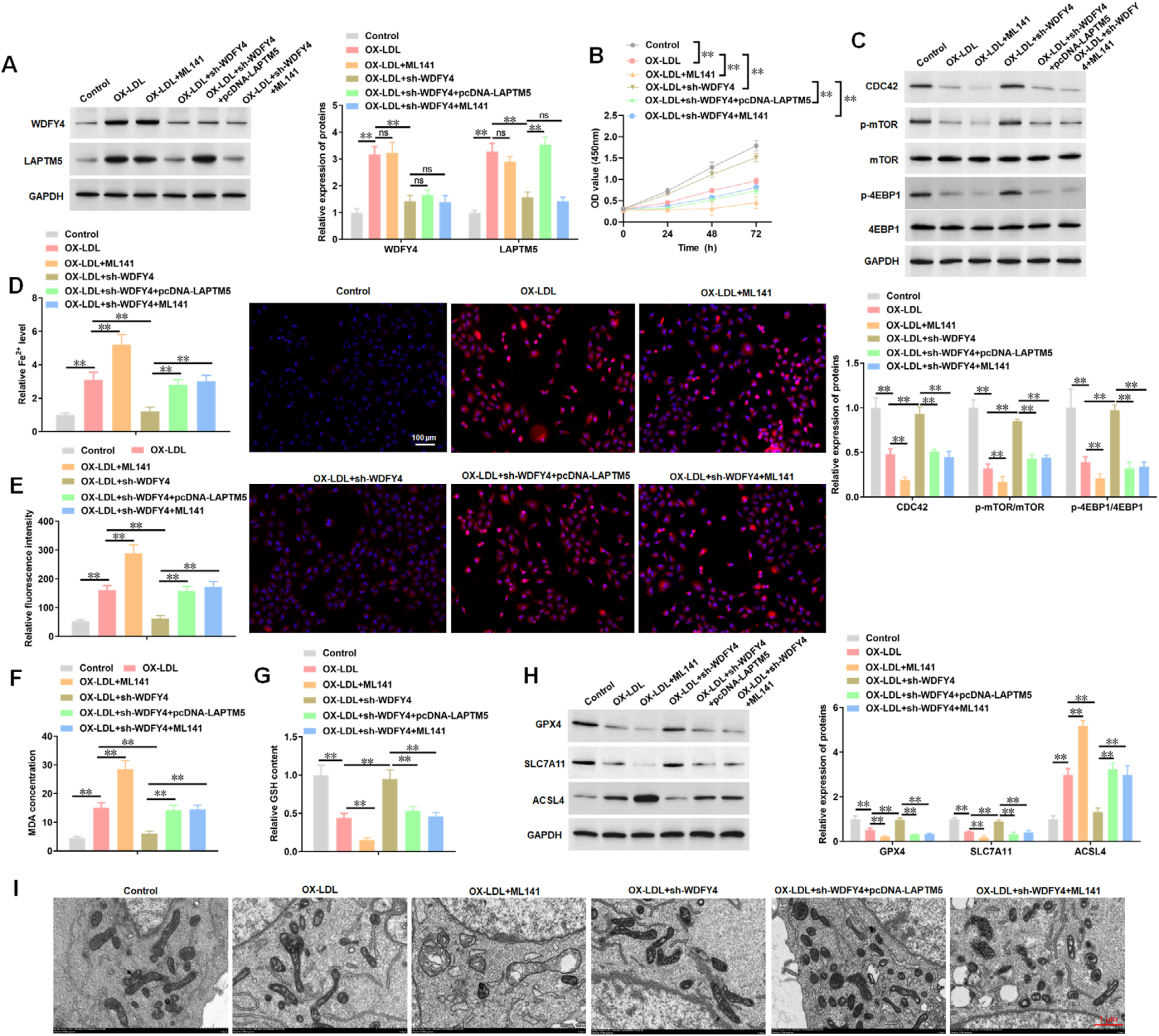
WDFY4 interacts with LAPTM5 and promotes ferroptosis in HAEC by inhibiting CDC42/mTOR/4EBP1/SLC7A11 pathway. After HAECs were treated with 100 μg/mL ox‐LDL for 24 h. ox‐LDL treated HAECs were transfected with sh‐WDFY4 and pcDNA‐LAPTM5 or treated with 10 μM ML141. (A) Western blotting was used to detect the expression level of WDFY4 protein in HAECs. (B) CCK‐8 was used to detect the activity of HAECs. (C) Western blotting was used to detect the expression level of CDC42, mTOR and 4EBP1 protein in HAECs. (D) ELISA was used to detect Fe^2+^ content in HAECs. (E) The level of lipid ROS in HAEC was detected by DCFH‐DA fluorescent probe method. Scale bar, 100 μm. (F) MDA content was detected with MDA assay kit. (G) GSH level was detected with GSH assay kit. (H) Western blotting was used to detect the level of ferroptosis‐related proteins. (I) Representative transmission electron microscopy pictures of mitochondria ultrastructure in HAECs were showed. Scale bar, 1 μm. *n* = 4. One‐way ANOVA or Two‐way ANOVA was used for comparison between multiple groups. **p < 0.01, and ns means non‐significant.

Previous studies have shown that LAPTM5 promotes CDC42 degradation via direct interaction [29], and LAPTM5 downregulation activates the mTOR pathway [30]. Given that CDC42 regulates mTOR signalling [31] and the mTOR/4EBP1 axis drives SLC7A11 protein synthesis [32], we hypothesised that WDFY4 modulates HAECs ferroptosis via the LAPTM5/CDC42/mTOR/4EBP1/SLC7A11 pathway. Western blot analysis revealed that the CDC42 inhibitor ML141 did not alter WDFY4 or LAPTM5 expression in ox‐LDL‐treated HAECs (Figure 5A). In ox‐LDL‐treated HAECs, WDFY4 knockdown reduced both WDFY4 and LAPTM5 levels, while LAPTM5 overexpression rescued the suppression of LAPTM5 expression in WDFY4‐silenced cells (Figure 5A). CCK‐8 assays showed that ML141 exacerbated ox‐LDL‐mediated cytotoxicity, whereas WDFY4 knockdown attenuated this effect. Notably, LAPTM5 overexpression or ML141 co‐treatment reversed the protective effect of WDFY4 deficiency on cell viability (Figure 5B). Furthermore, ox‐LDL treatment suppressed the expression of CDC42, p‐mTOR and p‐4EBP1, an effect that was amplified by ML141 (Figure 5C). In contrast, WDFY4 knockdown reversed the ox‐LDL‐induced suppression of these proteins (Figure 5C). The effects of WDFY4 knockdown on CDC42, p‐mTOR and p‐4EBP1 expression in ox‐LDL‐treated cells were abolished by either LAPTM5 restoration or ML141 administration (Figure 5C). In addition, ML141 aggravated the induction of ferroptosis by ox‐LDL in HAECs, which was mainly manifested by a significant increase in Fe^2+^ level (Figure 5D), lipid ROS level (Figure 5E) and MDA concentration (Figure 5F) in HAECs and a significant decrease in GSH content (Figure 5G), GPX4 and SLC7A11 protein expression levels (Figure 5H). Knockdown of WDFY4 attenuated the ferroptosis‐promoting effect of ox‐LDL on HAECs, which was reversed by LAPTM5 overexpression or ML141 treatment (Figure 5D–H). To further determine the content of Fe^2+^, the utilisation of a Ferro‐Orange fluorescent probe showed that LAPTM5 overexpression or ML141 treatment weakened the inhibitory effect of WDFY4 knockdown on ox‐LDL‐induced HAECs ferroptosis (Figure S3). Consistently, TEM showed that ML141 administration further exacerbated ox‐LDL‐induced mitochondrial volume reduction and structural destruction in HAECs. LAPTM5 overexpression or ML141 treatment reversed the inhibitory effects of WDFY4 knockdown on mitochondrial damage (Figure 5I). Collectively, these findings validate our hypothesis that WDFY4 regulates ferroptosis in HAECs through the LAPTM5/CDC42/mTOR/4EBP1/SLC7A11 axis.

### 3.5 LAPTM5 and ML141 Reversed the Alleviation Effect of sh‐WDFY4 on Cell Injury and Inflammatory Response in AS Model

**FIGURE 6:**
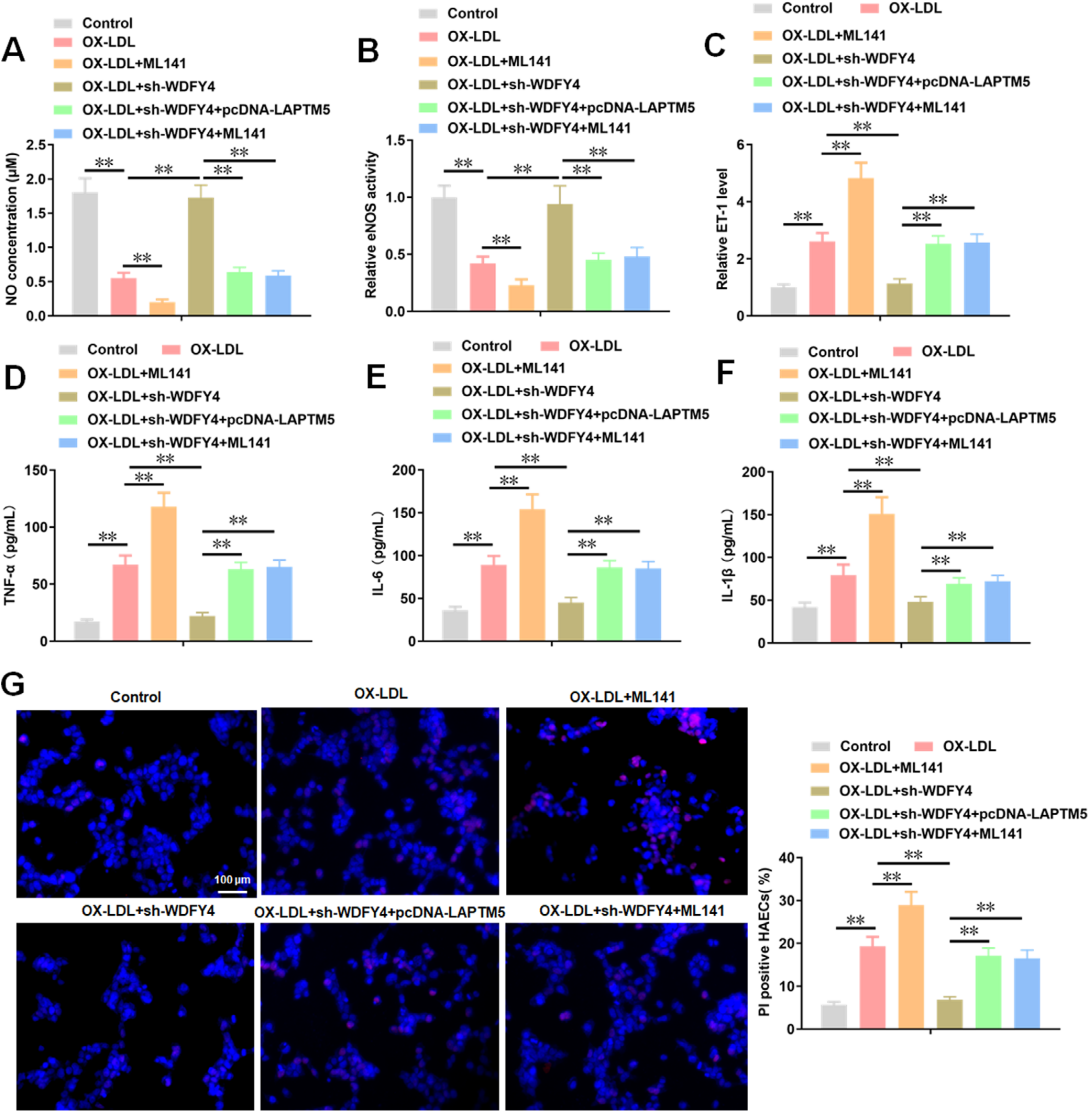
LAPTM5 and ML141 reversed the alleviation effect of sh‐WDFY4 on cell injury and inflammatory response in AS model cells. After HAECs were treated with 100 μg/mL ox‐LDL for 24 h. ox‐LDL treated HAECs were transfected with sh‐WDFY4 and pcDNA‐LAPTM5 or treated with 10 μM ML141. (A) The concentration of endothelial cell function marker NO in the supernatant of HAEC cells was detected by NO assay kit. (B) The activity of endothelial cell function marker eNOS in HAECs supernatant was detected by ELISA. (C) The level of endothelial cell function marker ET‐1 in the supernatant of HAECs was detected by ELISA. (D–F) The secretion level of inflammatory factors (TNF‐α, IL‐6 and IL‐1β) in the supernatant of HAECs was detected by ELISA. (G) PI staining was used to detect the mortality of HAECs treated with ox‐LDL. Scale bar, 100 μm. *n* = 4. One‐way ANOVA was used for comparison between multiple groups. **p < 0.01.

Building on the finding that WDFY4 regulates ferroptosis via the LAPTM5/CDC42/mTOR/4EBP1/SLC7A11 pathway, we investigated how WDFY4 mediates HAEC damage. We observed that treatment with ML141 exacerbated the damage caused by ox‐LDL to HAECs, which was manifested as a significant reduction in the NO concentration (Figure 6A) and eNOS activity (Figure 6B), along with a substantial increase in the ET‐1 level (Figure 6C) within HAECs. Knockdown of WDFY4 attenuated the inhibitory effect of ox‐LDL on NO concentration and eNOS activity in HAECs and the promoting effect on ET‐1 level, while these effects of sh‐WDFY4 on endothelial cell function markers could be reversed by the addition of LAPTM5 and ML141 (Figure 6A–C). For inflammatory factors, ML141 administration increased the secretion levels of TNF‐α, IL‐6 and IL‐1β in the supernatant of HAECs treated with ox‐LDL. LAPTM5 and ML141 restored counteracted the inhibitory effect of sh‐WDFY4 on the secretion of inflammatory factors (Figure 6D–F). PI staining results showed that ML141 promoted the death of HAECs treated with ox‐LDL. LAPTM5 and ML141 alleviated the inhibitory effect of sh‐WDFY4 on the death of ox‐LDL‐treated cells (Figure 6G). These results suggest that WDFY4 is co‐expressed with LAPTM5 and promotes HAECs injury and inflammation by inhibiting the CDC42/mTOR/4EBP1/SLC7A11 pathway.

### 3.6 Knockdown of WDFY4 and LAPTM5 Alleviated Ferroptosis‐Associated AS

**FIGURE 7:**
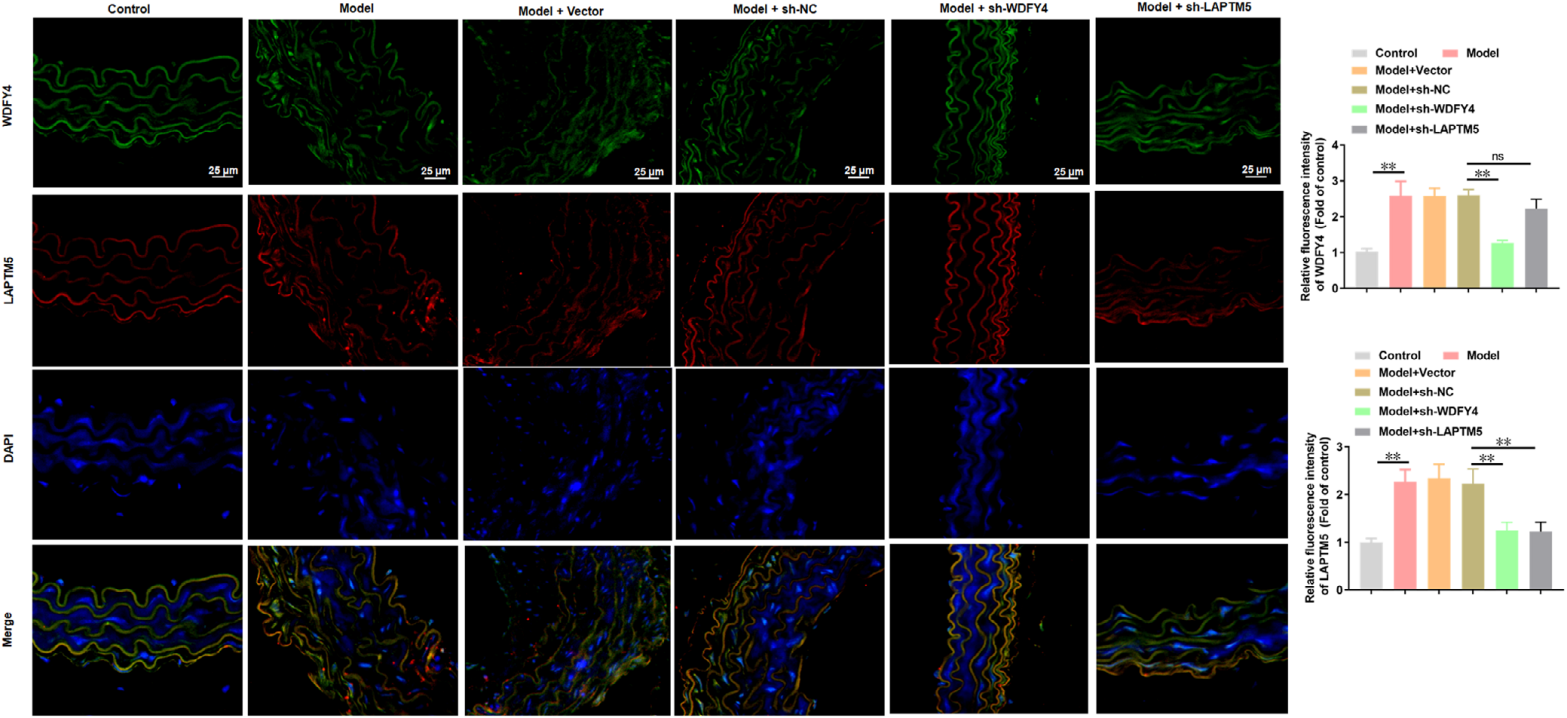
Fluorescence co‐localization of WDFY4 and LAPTM5 In vivo. ApoE^−/−^ mice were fed a high‐fat diet and injected with sh‐WDFY4, sh‐LAPTM5 or sh‐NC lentivirus every other day. Immunofluorescence staining of WDFY4 and LAPTM5 in the diseased area of the aortic root in mice were showed, and the fluorescence intensity was quantified. Scale bar, 25 μm. *n* = 8. One‐way ANOVA was used for comparison between multiple groups. **p < 0.01, and ns means non‐significant.

**FIGURE 8:**
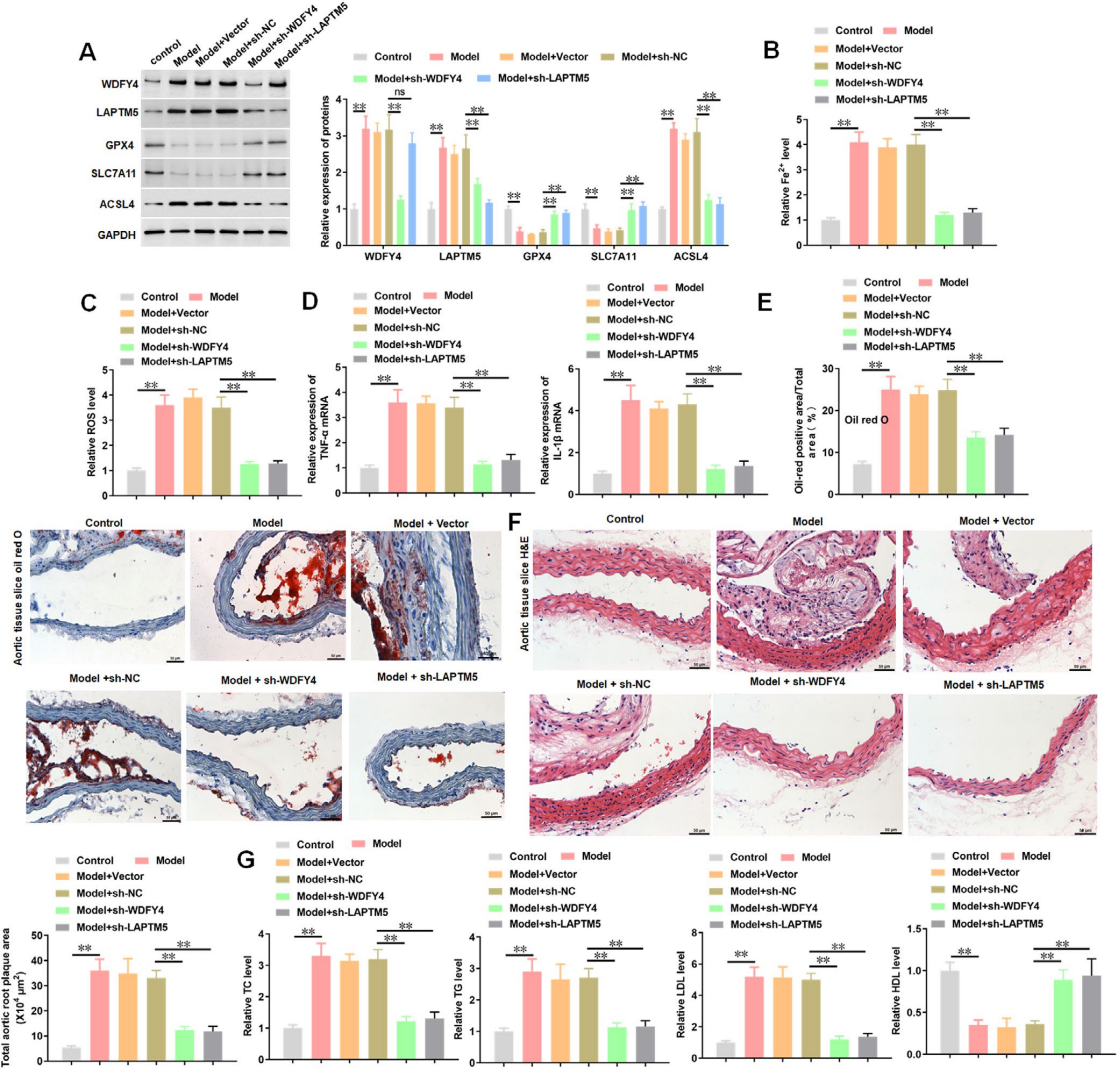
Knockdown of WDFY4 and LAPTM5 alleviates AS associated with ferroptosis. ApoE^−/−^ mice were fed a high‐fat diet and injected with sh‐WDFY4, sh‐LAPTM5 or sh‐NC lentivirus every other day. (A) Western blotting was used to detect the expression levels of WDFY4, LAPTM5 and ferroptosis‐related proteins in mouse aortic tissue. (B) ELISA was used to detect the Fe^2+^ level in serum of mice. (C) The level of ROS in serum of mice was detected. (D) The expression levels of inflammatory factors TNF‐α and IL‐1β mRNA in serum of mice were detected by qPCR. (E) Oil red O staining was used to detect the degree of arterial plaque. Scale bar, 50 μm. (F) HE staining was used for pathological analysis of arterial tissue. Scale bar, 50 μm. (G) The serum TC, TG, LDL and HDL levels were detected by ELISA. *n* = 8. One‐way ANOVA was used for comparison between multiple groups. **p < 0.01, and ns means non‐significant.

Finally, in vivo experiments were performed to verify the role of WDFY4 and LAPTM5 in ferroptosis‐associated AS. Double immunofluorescence staining showed that the atherosclerotic mice exhibited marked upregulation of WDFY4 and LAPTM5 expression, which was partially reversed by simultaneous WDFY4 knockdown (Figure 7). Furthermore, LAPTM5 knockdown reduced the expression of LAPTM5 in model mice, but showed no significant influence on WDFY4 expression (Figure 7). The results of Western blotting also confirmed these findings (Figure 8A). Compared with the Control group, model mice exhibited significant upregulation of ACSL4 protein expression, accompanied by marked downregulation of GPX4 and SLC7A11 (Figure 8A). Notably, administration of sh‐WDFY4 or sh‐LAPTM5 effectively attenuated ACSL4 upregulation and restored GPX4/SLC7A11 expression levels in the aorta of model mice (Figure 8A). The levels of Fe^2+^ and ROS were increased in model mice, whereas WDFY4 or LAPTM5 knockdown reversed these effects (Figure 8B,C). The results of qPCR showed that the expression levels of TNF‐α and IL‐1β mRNA in aortic tissue of model mice were significantly higher, while WDFY4 or LAPTM5 knockdown inhibited the expression levels of TNF‐α and IL‐1β mRNA in aortic tissue of model mice (Figure 8D). The results of oil red O staining and HE staining showed that compared with the control group, the lesion area, lipid accumulation, plaque and necrotic core area of AS in the model group were significantly increased, while administration of sh‐WDFY4 or sh‐LAPTM5 mitigated the development of AS plaque (Figure 8E,F). In addition, the levels of TC, TG and LDL in the serum of model mice were higher, and the level of HDL was lower. Both sh‐WDFY4 and sh‐LAPTM5 reduced serum TC, TG and LDL levels in model mice, and increased HDL levels (Figure 8G). In general, these results indicated that sh‐WDFY4 and sh‐LAPTM5 treatment can alleviate ferroptosis‐related AS.

To further explore the role of WDFY4 in AS development in mice, WDFY4^ECKO^ mice and their littermate WDFY4^WT^ mice were fed with a Western diet for 16 weeks after receiving an injection of PCSK9 AAV through tail veins. At the end of the experiment, no significant differences in body weight, blood glucose or the levels of serum TC, TG, HDL‐C or LDL‐C were observed between WDFY4^ECKO^ mice and WDFY4^WT^ littermates (Figure S4A–F). Immunofluorescence staining of the atherosclerotic aorta showed a specific ablation of WDFY4 in the ECs of WDFY4^ECKO^ mice compared to WDFY4^WT^ control mice (Figure S5A). The significantly reduced WDFY4 expression was confirmed in ECs of EC‐WDFY4 deletion mice by RT‐qPCR (Figure S5B) and Western blotting (Figure S5C). Notably, Oil Red O staining showed a significant increase in atherosclerotic plaque area in both male and female WDFY4^ECKO^ mice compared to WDFY4^WT^ mice (Figure S5D). Histological analysis of the aortic root demonstrated that depletion of endothelial WDFY4 decreased atherosclerotic plaque size (Figure S5E). Furthermore, we also found that WDFY4^ECKO^ mice exhibited obviously reduced serum TNF‐α and IL‐1β levels compared with WDFY4^WT^ control mice (Figure S5F,G). We then evaluated the ROS level in the aortic root of mice and observed a significant decrease in the ROS levels in WDFY4^ECKO^ mice compared with the littermate WDFY4^WT^ mice (Figure S5H). Furthermore, TEM revealed that the deficiency of WDFY4 significantly alleviated mitochondrial damage and the mitochondrial structure was basically restored compared with the control group (Figure S5I). As expected, depletion of WDFY4 significantly promoted the protein expression of GPX4 and SLC7A11, and inhibited the expression of ACSL4 protein in the aorta (Figure S5J). Consistent with the results in vitro, the expression levels of LAPTM5, CDC42, p‐mTOR and p‐4EBP1 were reduced in WDFY4^ECKO^ mice (Figure S5K). Taken together, these findings further demonstrated that WDFY4 deficiency exerts anti‐atherosclerotic effects by regulating the LAPTM5/CDC42/mTOR/4EBP1/SLC7A11 signalling pathway to suppress ferroptosis.

## 4 Discussion

Although ferroptosis is widely accepted as a risk factor for AS, its underlying mechanism is not fully understood. Here, we confirmed that the expression of WDFY4 was increased in both the AS cell model and the animal model. Downregulation of WDFY4 can reduce endothelial cell damage and alleviate AS progression by inhibiting the inflammatory response and ferroptosis. Mechanistically, WDFY4 interacts with LAPTM5 and promotes LAPTM5 expression to enhance ferroptosis in HAECs. Further investigations reveal that WDFY4 co‐expresses with LAPTM5 and exacerbates ferroptosis by inhibiting the CDC42/mTOR/4EBP1/SLC7A11 pathway, thereby inducing endothelial injury and inflammation. In addition, in vivo experiments using ApoE^−/−^ mice and WDFY4‐knockout (WDFY4^ECKO^) transgenic mice both confirmed that WDFY4 deficiency mitigates ferroptosis and suppresses AS plaque formation. Collectively, these findings establish WDFY4 as a key pro‐atherosclerotic factor and highlight WDFY4 inhibition as a potential therapeutic strategy for AS.

WDFY4, a large protein of unknown function primarily expressed in immune tissues [33], has been reported that WDFY4 is a susceptibility gene for a variety of autoimmune diseases, such as lupus erythematosus, primary biliary cholangitis, idiopathic arthritis and type 1 diabetes [19, 20, 34]. WDFY4 deficiency in non‐obese diabetic mice improved autoimmune diabetes and insulitis [16]. It has also been found that WDFY4 deficiency in mice with lupus erythematosus was effectively alleviated [17]. In addition, the expression of WDFY4 is also associated with rapid progressive interstitial lung disease (RPILD), and high levels of WDFY4 were found in RPILD [35]. It has been reported that WDFY4 could enhance MDA5‐induced apoptosis via augmenting activation of NK‐κB [36]. TLR4/NF‐κB signalling is essential for the transmission of inflammatory signals, while ox‐LDL can be directly recognised by TLR4 and activate NF‐κB. All this evidence suggested that WDFY4 may play a critical role in ox‐LDL‐induced inflammation and damage in endothelial cells. Furthermore, inflammatory infiltration is one of the most salient pathological features of atherosclerosis in both animal models and human studies. In this study, in vitro silencing of WDFY4 in HAECs reduced ox‐LDL‐induced secretion of IL‐6, IL‐1β and TNF‐α. In vivo experiments showed that depletion of WDFY4 manifested with decreased expression of IL‐1β and TNFα in the aortic tissues and in the serum, indicating an attenuated inflammatory response in AS mice with WDFY4 knockdown or knockout. Additionally, WDFY4^ECKO^ mice resulted in lower co‐localisation with CD31 endothelial cells in blood vessels and exhibited smaller and more stable atherosclerotic lesions than littermate WDFY4^WT^ mice. WDFY4 was reported to be highly associated with AS factors like immune responses [24] and also induction of HAECs injury obtained from our present study, which may partly promote such atherosclerotic changes. Importantly, endothelium‐derived NO that is regulated by eNOS also acts as an antioxidant to inhibit LDL oxidation and peroxidases [37]. In the current study, ox‐LDL treatment resulted in a substantial reduction in eNOS activity and NO levels in HAECs, but this trend was reversed by WDFY4 knockdown in HAECs. These findings further indicated that WDFY4 could induce vascular endothelial inflammatory injury, which may continuously promote the initiation of AS.

Ferroptosis is associated with iron and lipid metabolism, and it plays a pathological role in AS by linking to oxidative stress [38]. Ferroptosis of endothelial cells, macrophages and vascular smooth muscle cells is also involved in the occurrence and progression of AS [39, 40]. Inhibition of ferroptosis alleviates AS by reducing lipid peroxidation and preserving endothelial function [41, 42]. It has also been found that induction of ferroptosis in macrophages and endothelial cells exacerbates AS progression [43, 44]. This study explored the mechanism of WDFY4 affecting atherosclerosis. Our experiments revealed that WDFY4 activated ferroptosis in vascular endothelial cells by triggering accumulation of ROS, iron accumulation and lipid peroxidation, which are typical features of ferroptosis. As mitochondria play an essential role in ferroptosis, we also noticed that knockdown of WDFY4 weakened ox‐LDL‐induced mitochondrial volume reduction and structural destruction in HAECs. GSH depletion has been shown to affect GPX4 activity and stability, thus causing cells to be more sensitive to ferroptosis and participating in AS progression [45]. In the present study, we observed that WDFY4 depletion increased intracellular GSH levels and promoted GPX4 expression at the same time in ox‐LDL‐treated HAECs. Overall, we revealed that WDFY4 activates ferroptosis to exacerbate endothelial inflammation injury and ultimately accelerates the development of AS.

LAPTM5, a lysosomal membrane protein with five transmembrane domains [46], negatively regulates B cell activation [47] but promotes macrophage pro‐inflammatory signalling [48]. After ICH, the knockdown of LAPTM5 improved neuronal degeneration and inflammatory cascades after secondary brain injury [49]. Silencing LAPTM5 significantly down‐regulated the mRNA expression of inflammatory factors in PA‐treated HK2 cells [50]. In addition, the accumulation of LAPTM5 in nerve cells led to cell death [51]. LAPTM5 is also closely related to cardiovascular diseases. For example, LAPTM5, as a hub gene of AS, is highly expressed in AS plaques [28]. In addition, LAPTM5 is a potential diagnostic marker for left ventricular hypertrophy (LVH) in hypertension, and its expression in LVH is significantly higher than that in normal controls [52]. Mechanistically, LAPTM5 shows a strong correlation with ROS, autophagy and other markers. LAPTM5 promotes intrinsic autophagy flux by promoting the formation of autophagic lysosomes [53, 54]. Similar to the results of these studies, we found that the expression of LAPTM5 was increased in both the AS cell model and animal models, where it physically interacted with WDFY4. In addition, LAPTM5 overexpression reversed the inhibitory effects of WDFY4 knockdown on HAECs ferroptosis induced by ox‐LDL, which was mainly manifested by a significant increase in Fe^2+^ level, lipid ROS level and MDA concentration in HAECs, and a significant decrease in GSH content, GPX4 and SLC7A11 protein expression levels. Gain‐of‐function experiments revealed that LAPTM5 overexpression exacerbated ox‐LDL‐induced cellular damage and inflammatory cytokine release in WDFY4‐silenced HAECs. In vivo interference with LAPTM5 substantially ameliorated pathological progression in AS model mice, evidenced by reduced plaque area and attenuated aortic inflammation. These findings further supported LAPTM5 as a potential biomarker for AS that was associated with inflammatory reactions [55].

Previous studies have shown that LAPTM5 promotes CDC42 degradation through direct interaction [29], while LAPTM5 downregulation activates the mTOR pathway [30]. CDC42 is reported to regulate blood lipids, atherosclerosis and inflammation. In addition, CDC42 functioned as an upstream regulator of the mTOR pathway [31], and the mTOR/4EBP1 axis drives the synthesis of SLC7A11 protein [32]. However, the coordinated role of these four components in a unified regulatory system remained uncharacterised. In this study, we found that interference with WDFY4 promoted the expression of CDC42, p‐mTOR, p‐4EBP1 and SLC7A11, indicating WDFY4 regulates the mTOR/4EBP1 pathway and decreased the expression of SLC7A11 protein level. Gain‐of‐function experiments revealed that LAPTM5 overexpression or CDC42 inhibition attenuated the suppressive effects of WDFY4 knockdown on inflammation and ferroptosis in ox‐LDL‐treated HAECs.

In summary, this study demonstrates that WDFY4 drives HAECs ferroptosis and accelerates AS progression, and identifies the molecular mechanism WDFY4‐mediated endothelial injury involves the inhibition of the CDC42/mTOR/4EBP1/SLC7A11 pathway. Our findings suggest that targeting WDFY4 may be beneficial for slowing the progression of atherosclerotic disease.

## Author Contributions

**Nier Zhong:** conceptualization (equal), investigation (equal), methodology (equal), supervision (equal), writing – original draft (equal), writing – review and editing (equal). **Xiting Nong:** conceptualization (equal), investigation (equal), methodology (equal), supervision (equal), writing – original draft (equal), writing – review and editing (equal). **Guang Yang:** data curation (equal), methodology (equal), resources (equal), software (equal), validation (equal), visualization (equal), writing – review and editing (equal).

## Ethics Statement

This study was approved by the Ethics Committee of Shaanxi Provincial People's Hospital (Approval number: 2023 K‐S147).

## Consent

The authors have nothing to report.

## Conflicts of Interest

The authors declare no conflicts of interest.

## Supporting information


Figure S1.

Figure S2.

Figure S3.

Figure S4.

Figure S5.

Data S1.

## Data Availability

The datasets generated during the current study are available from the corresponding author on reasonable request.
