# Supplementary figures and images for: WDFY4 Promotes the Progression of Atherosclerosis by Regulating Ferroptosis Mediated by the LAPTM5/CDC42/mTOR/4EBP1/SLC7A11 Pathway
Source: J Cell Mol Med. 2025 Aug 3;29(15):e70729. doi: 10.1111/jcmm.70729 (PMC12319153; doi:10.1111/jcmm.70729)

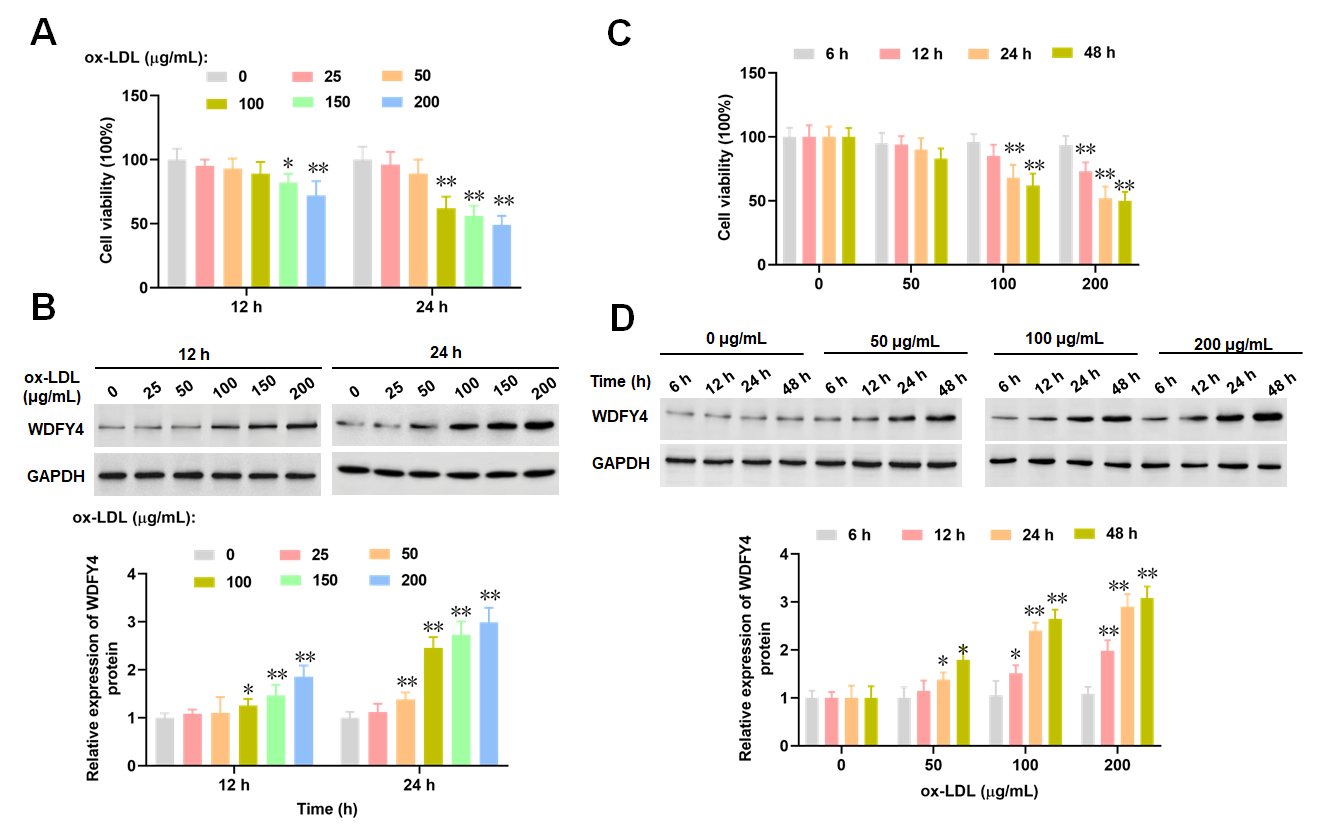

Supplement: Supplementary file 1 — Figure S1. [file JCMM-29-e70729-s004.tif]

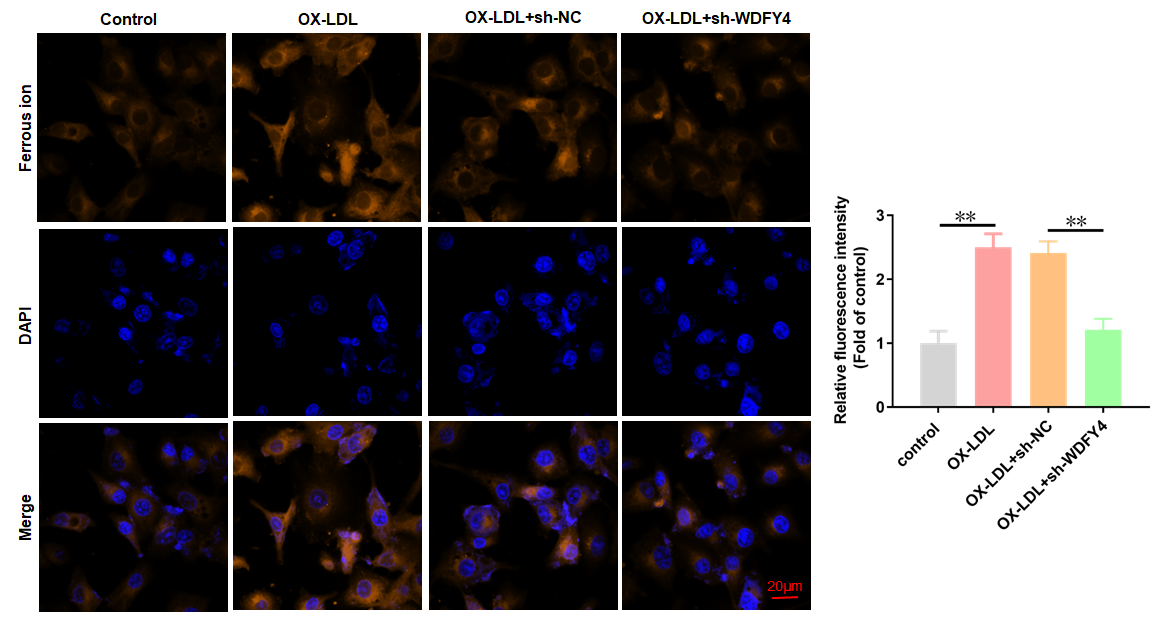

Supplement: Supplementary file 2 — Figure S2. [file JCMM-29-e70729-s005.tif]

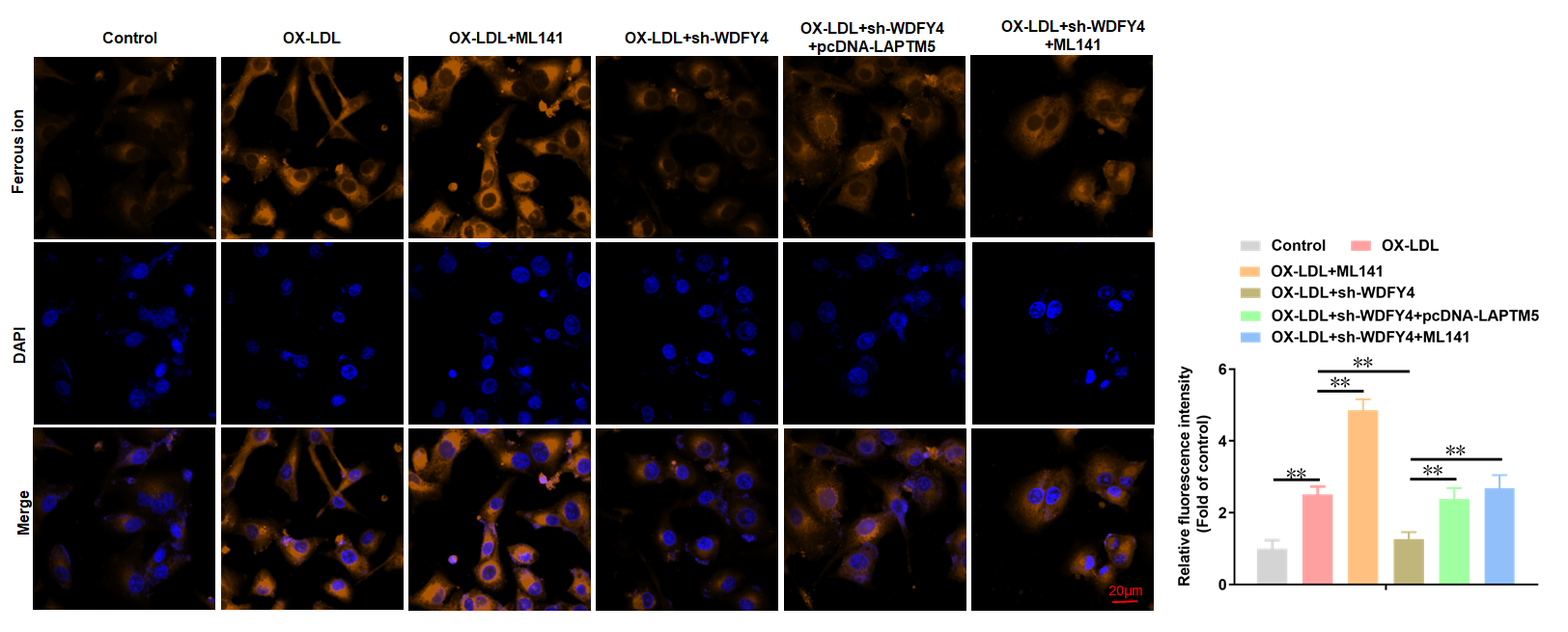

Supplement: Supplementary file 3 — Figure S3. [file JCMM-29-e70729-s001.tif]

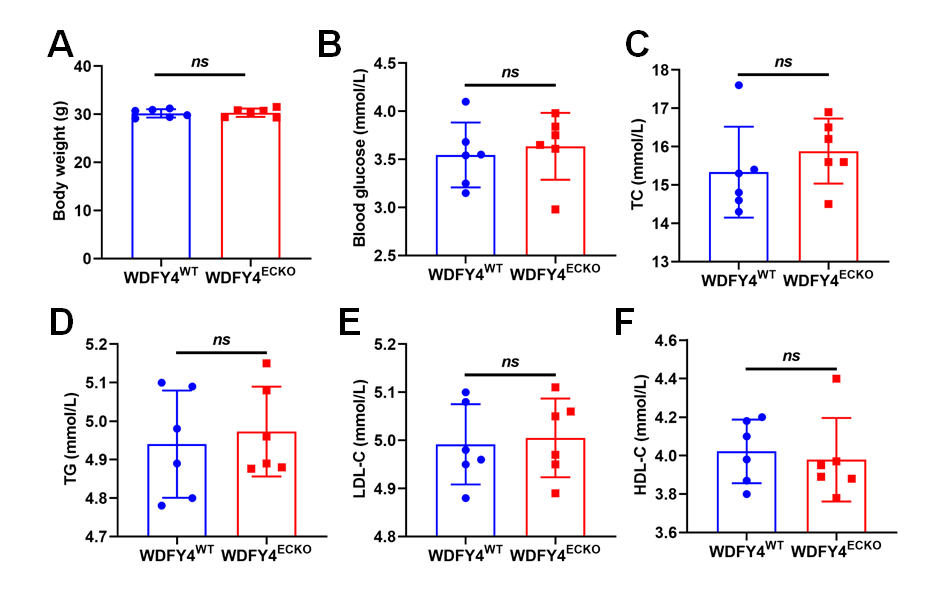

Supplement: Supplementary file 4 — Figure S4. [file JCMM-29-e70729-s003.tif]

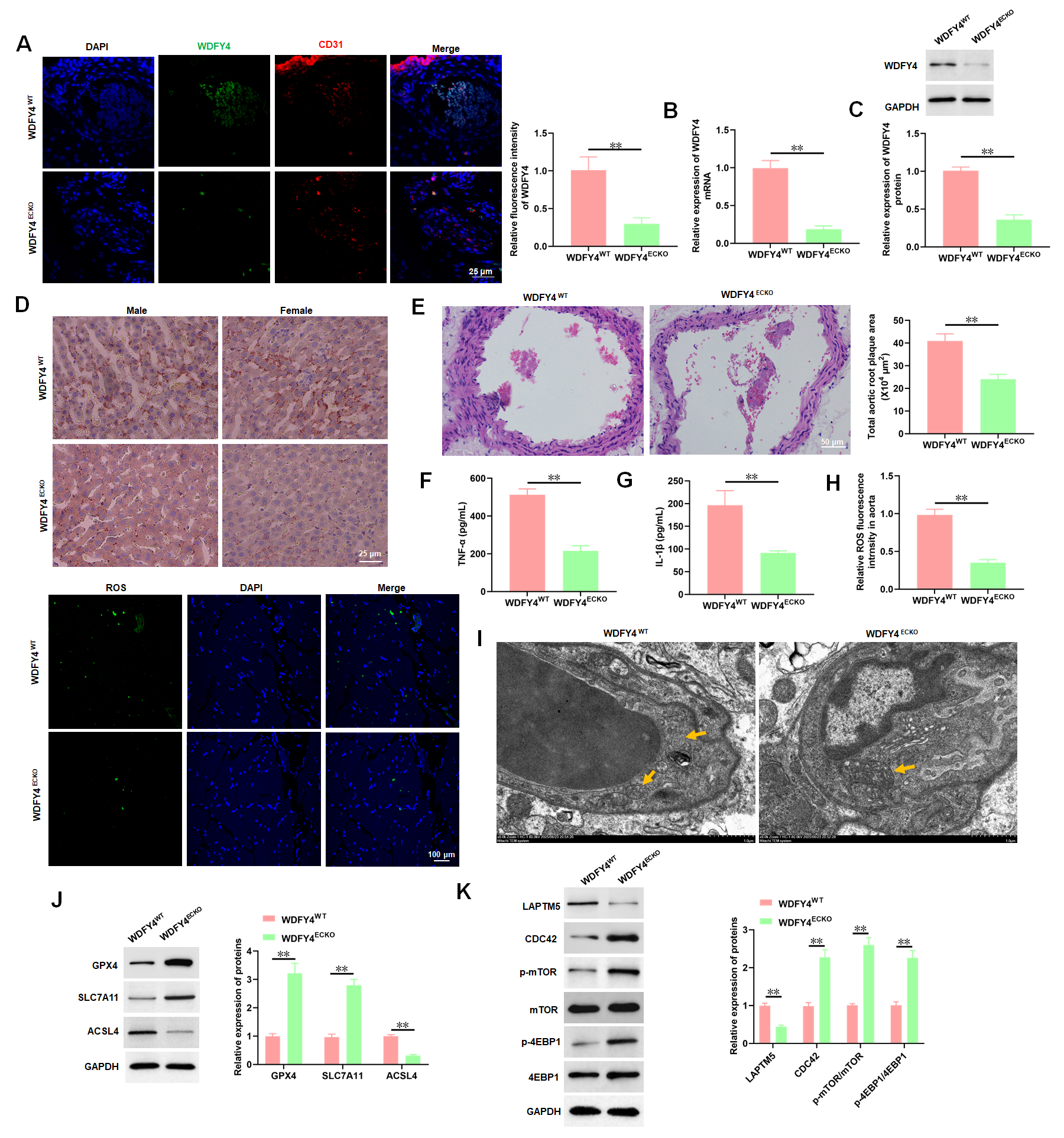

Supplement: Supplementary file 5 — Figure S5. [file JCMM-29-e70729-s002.tif]
