# Supplementary material for: WDFY4 Promotes the Progression of Atherosclerosis by Regulating Ferroptosis Mediated by the LAPTM5/CDC42/mTOR/4EBP1/SLC7A11 Pathway
Source: J Cell Mol Med. 2025 Aug 3;29(15):e70729. doi: 10.1111/jcmm.70729 (PMC12319153; doi:10.1111/jcmm.70729)
Supplement: Supplementary file 6 — Data S1. [file JCMM-29-e70729-s006.doc]

**Supplemental figures**

**Supplemental figure 1** **WDFY4 is up-regulated in ox-LDL-treated HAEC and inhibits cell viability.** A, B. HAECs were treated with ox-LDL (0, 25, 50, 100, 150, and 200 μg/mL) for 12 or 24 h. The effects of different concentrations of ox-LDL at 12 h and 24 h on the cell viability of HAECs and the expression levels of WDFY4 protein were detected by CCK-8 and Western blotting. C, D. HAECs were treated with ox-LDL (0, 50, 100, or 200 μg/mL) over an extended timeframe (6, 12, 24, and 48 h). Cell viability and the expression levels of WDFY4 protein were detected by CCK-8 and Western blotting. n = 4. One-way ANOVA was used for comparison between multiple groups. Data are presented as mean ± SD. * *P* < 0.05 and ** *P* < 0.01.

**Supplemental figure 2 Knockdown of WDFY4 inhibits ox-LDL-induced the increase of Fe^2+^ content in HAECs.** Representative images of intracellular iron fluorescence by FerroOrange probe, and representative quantification of ferrous ion levels were displayed. Scale bar, 20 μm. n = 4. One-way ANOVA was used for comparison between multiple groups. Data are presented as mean ± SD. ** *P* < 0.01.

**Supplemental figure 3 LAPTM5 overexpression or ML141 treatments reversed the effects of WDFY4 knockdown on Fe^2+^ content in ox-LDL-induced HAECs.** Representative images of intracellular iron fluorescence by FerroOrange probe, and representative quantification of ferrous ion levels were displayed. Scale bar, 20 μm. n = 4. One-way ANOVA was used for comparison between multiple groups. Data are presented as mean ± SD. ** *P* < 0.01.

**Supplemental figure 4 Analysis of body weight and serum biochemistry in WT or EC-specific WDFY4 knockout mice.** Analysis of body weight (A), blood glucose (B), TC (C), TG (D), LDL-C (F), and HDL-C (F) levels were showed. n = 8. Student’s t test was used for comparison between the two groups. Data are presented as mean ± SD. ns means non-significant.

**Supplemental figure 5 EC-specific knockout of WDFY4 alleviates atherosclerosis in mice.** A. Immunofluorescence staining of WDFY4 and CD31 in the diseased area of the aortic root in WDFY4^ECKO^ mice was performed. Scale bar, 25 μm. B, C. The expression of WDFY4 mRNA and protein levels was detected by qRT–PCR and Western blotting. D. Representative images of atherosclerotic lesions in the aortic root stained with Oil Red O in mice were showed. Scale bar, 25 μm. E. HE staining of aortic root lesions was performed for pathological analysis. Scale bar, 50 μm. F, G. The contents of plasma TNF-α and IL-1β levels were detected by ELISA. H. The level of lipid ROS was detected by DCFH-DA fluorescent probe method. Scale bar, 100 μm.I. Representative transmission electron microscopy pictures of mitochondria ultrastructure. Scale bar, 1 μm. J. Western blotting was used to detect the expression levels of ferroptosis-related proteins in mice aortic tissue. H. Western blotting was used to detect the expression level of LAPTM5, CDC42, mTOR and 4EBP1 protein in mice aortic tissue. n = 8. Student’s t test was used for comparison between the two groups. ** *P* < 0.01.
